# Editorial: A view of cell migration dynamics at the single-cell level

**DOI:** 10.3389/fcell.2023.1348039

**Published:** 2023-12-12

**Authors:** Claudia Tanja Mierke

**Affiliations:** Faculty of Physics and Earth Systems Science, Peter Debye Institute of Soft Matter Physics, Biological Physics Division, Leipzig University, Leipzig, Germany

**Keywords:** individual migration, topotaxis, aerotaxis, cell density, triple negative breast cancer, mitochondria, cell pot model, corticogenesis

A View of Cell Migration Dynamics at the Single-Cell Levels represents a specialized Research Topic (RT) in the Section Cell Adhesion and Migration of the Journal for Frontiers in Cell and Developmental Biology Research, as it covers four really intriguingly original research articles. In the past, the migration dynamics of single cells have been investigated in a number of studies (Mierke et al., 2011; Mierke et al., 2022; Mierke, 2013; Chen et al., 2015; Kunschmann et al., 2019; Wang and Yao, 2020), but there is much more to explore, as the RT manuscripts show. The first manuscript is focused on the biomaterial preparation and its usage in cellular analysis. Therein, the Shivani et al. explore the effect of surface roughness on migration and directionality of movement using human MG63 osteosarcoma cells. The second original research article emphasizes the lack of classification options in Triple-Negative Breast Cancers (TNBCs). Burger et al. therefore devised realistic spatial simulations with the use of a Cellular Potts model (CPM) that provided an in-depth analysis of the pseudopod dynamics. After comparing the simulation data with the experimental data, the authors proposed a mechanistic view of density-dependent cell migration characteristics and the clustering process. The third original research article explores the aerotaxis of cells. Thereby, Hirose et al. uses *Dictyostelium discoideum* as a model system of cell motility to investigate the effect of oxygen on individual cell migration. The fourth original research article examines the mechanisms of corticogenesis in a mouse model centered on single-cell RNA-seq (scRNA-seq) transcriptome datasets derived from mouse and human fetal cortexes (Zhou et al.). All four manuscripts are outstanding and of great importance and of interest to a wide audience of scientists.

Extracellular matrix communication is among the key environmental drivers of cell migration, such as individual cell migration, which include stiffness-dependent durotaxis and adhesion-dependent haptotaxis. Shivani et al. investigate in their original research article the effect of contact guidance, such as the roughness based topotaxis. In contrast to previous research on topotaxis, where standard photolithography was typically implemented to fabricate micrometer- or submicrometer-scale features with the same height and varying spatial density, the authors propose a new technique for the program-controlled production of substrates with varying surface roughness characteristics by two-photon polymerization. A surface roughness of 0.29–1.11 μm can be generated by regulating the voxel spacing between neighboring hardened ellipsoidal voxels. Ormocomp^®^ patterns are transferred to polypropylene sheets for cell migration experiments utilizing the nanoimprinting technique. Their experimental findings imply that MG63 cells can perceive the spatial pattern of the subjacent extracellular roughness and are capable of adjusting their migration speed and migration direction. The cells exhibited three different types of cellular characteristics. Firstly, cells move faster on surfaces with higher roughness. Secondly, the cells migrated preferentially from areas with higher roughness to areas with lower roughness, and their migration speed likewise diminished with a decrease in roughness. Thirdly, the migration rate in the lower roughness area stayed unaltered on a stepped surface with a steeper roughness. Consequently, the steepness of the roughness gradient appears to be an additional signal for the microenvironment alongside the surface roughness. The advanced 3D lithography method, two-photon polymerization, therefore provided a high-precision tool for manufacturing substrates with programmable topography in the micrometer and sub-micrometer range. In conclusion, the fusion of two-photon polymerization and nanoimprint methods has the potential to be a novel manufacturing technique to improve the 3D complex structures for the exploration of topotactic cell migration.

The classification of breast cancer relies largely on the status of three different receptors: ER (estrogen receptor), PR (progesterone receptor), and human epidermal growth factor receptor 2 (HER2). The classification of breast cancers (BCs) without these three receptors, which are termed Triple-Negative Breast Cancers (TNBCs), needs to rely on other criteria. Claudin-deficient BC, a type recently relabeled as a BC phenotype instead of an intrinsic subtype (Fougner et al., 2020), is typified by accumulation of epithelial-mesenchymal transition (EMT) signatures and stem cell-like traits (Prat et al., 2010). The capacity of cancer cells to disseminate from primary tumors into adjacent tissues is an established feature of metastatic fate. The quantification of cell migration traits like migration velocity and persistence aids in gaining an insight into the prerequisites for this kind of invasiveness. A factor that may impact invasion is the way in which local cancer cell density determines the features of cell migration, which Burger et al. address through a coupled experimental and computational modeling effort. Initially, the authors acquired and analyzed time-lapse imaging results of two aggressive TNBC cell lines, namely HCC38 and Hs578T, performing 2D migration assays at varying cell densities. HCC38 cells showed a counterintuitive rise in velocity and persistence with rising density, unlike Hs578T, which did not. In addition, HCC38 cells showed strong clustering with active pseudopod-driven migration, particularly at low density, while Hs578T cells kept a scattered localization. For a mechanistic comprehension of density-dependent cell migration features and clustering, the authors performed realistic spatial simulations based on a CPM containing an overt characterization of the pseudopod dynamics. CPM constitutes a spatial grid-based constraint formalism for analyzing the spatio-temporal performance of biological cell populations. The method can be implemented if the particulars of the intercellular crosstalk are substantially governed by the shape and size of the individual cells and the length of the interface between contiguous cells. The CPM analysis showed that pseudopods applying traction to the cell and engaging with the pseudopod tips through enhanced adhesion could account for the experimentally seen rise in velocity and persistence with rising density in HCC38 cells. Consequently, density-dependent migration characteristics are an emerging characteristic of single cell traits with no additional mechanisms required. Finally, this signifies that the dynamics and the interplay of pseudopods may contribute to the aggressive character of cancers by facilitating their propagation.

The oxygen environment’s spatial and temporal fluctuations influence the way in which different cells react and are implicated in physiological and pathological processes. The ability of *Dictyostelium* to move towards extracellular cAMP has been thoroughly characterized and became a powerful model for chemotaxis in various organisms (Pal et al., 2019). cAMP functions to regulate actin polymerization and subsequently cell motility via G-protein-coupled receptors (GPCR). Spatial gradients of cAMP across the anterior and posterior part of cells are identified and augmented by second messengers intracellularly to control movement. Hirose et al., who have previously conducted research using *Dictyostelium discoideum* as a cellular motility model system, have found that aerotaxis towards an oxygen-rich area below 2% O_2_. While aerotaxis of *Dictyostelium* appears to be an important tactic for finding what is needed for survival, the mechanism behind this phenomenon is still far from certain. It is hypothesized that an oxygen concentration gradient creates a secondary oxidative stress gradient that drives cell migration toward a higher oxygen level. This type of mechanism has been hypothesized, but not entirely proven, to account for aerotaxis of human cancer cells. The authors focused on the involvement of flavohemoglobins in aerotaxis, which are proteins that can function as potential oxygen sensors as well as regulators of nitric oxide and oxidative stress. The migration characteristics of *Dictyostelium* cells were monitored using time-lapse phase-contrast microscopy under both self-induced and forced oxygen gradients, whereby also the effect of chemicals that produce or impede oxidative stress was explored. The findings suggest that both oxidative and nitrosative stress are not engaged in the aerotaxis of *Dictyostelium*, instead having cytotoxic impacts that are intensified by hypoxia. Since mitochondria are important oxygen sources and may be implicated in direct or indirect oxygen scavenging for aerotaxis, however, the authors found that mitochondria are not required for aerotaxis. As a consequence, mitochondria are excluded to fulfill a direct function as oxygen sensors for *Dictyostelium*.

The cerebral cortex development demands various biological mechanisms culminating in the development of functional neural networks. In mammals, the cortical progenitors can differentiate to a broad-spectrum of cell types, such as neurons and non-neuron cells. Consequently, six neocortical layers were created (Franchini, 2021). The mechanisms underpinning corticogenesis are presently in the process of being determined. Zhou et al. obtained the most extensive single-cell RNA-seq (scRNA-seq) datasets from mouse and human fetal cortexes for analysis and validated the findings using co-immunostaining approaches. By dissecting developmental trajectories using scRNA-seq data sets in mice, the authors pinpointed a distinct developmental subpathway involving a population of cells expressing both deep and upper layer neurons (DLNs and ULNs) with specific biomarkers that were present at E13.5 but lacking in adults. In this population of cells, the proportion of cells expressing DLN and ULN biomarkers declined and enhanced, respectively, throughout development, pointing to a straightforward neuronal transition, which is referred to as D-T-U. Genes that were found to be markedly highly/uniquely expressed in the D-T-U cell population appeared to be substantially increased in PTN/MDK signaling cascades linked to migration. Both results were verified using co-immunostaining with DLNs, ULNs and D-T-U specific biomarkers at several time points. Moreover, six genes, which are co-expressed in mice with D-T-U specific biomarkers, that exhibit potentially opposing temporal expression between humans and mice throughout fetal cortical development have been linked to neuronal migration and cognitive performance. Neurons from various layers of the adult prefrontal cortex (PFC) expressed D-T-U-specific genes in both humans and mice. In summary, the authors identified a distinct cell population D-T-U that exhibits a direct neuronal shift from DLNs to ULNs and migration in mice throughout fetal cortical development. This discrepancy may be related to the differences in cortical evolution seen in humans and mice.

All four original research articles deal with single cell migration on vastly different cellular systems, but finally they all contribute to the importance of analyzing the dynamical nature of single cell migration. The combination of many different methods and techniques from mostly different disciplines makes it possible to get a complete overview on single cell migration dynamics and to recognize similarities and differences. There are many more interesting facts to explore in the field of single cell migration dynamics, such as whether other factors from the environment of the cells under investigation have an influence here and the effect of the dimensionality of the investigation system on the migration behavior. In future, a special focus should be placed on the development of mechanically tunable biomaterials that are placed in a 3D microenvironment to investigate the migration of individual cells in greater depth.
